# Diacetyl-induced bronchiolitis obliterans involves ubiquitin C upregulation and fibrosis-related gene activation in rats

**DOI:** 10.3389/fphar.2025.1671557

**Published:** 2025-11-28

**Authors:** Hailian Wang, Wen Jiang, Junchao Xiao, Yan Geng, Qing Ye, Wei Ge

**Affiliations:** 1 The Department of Pediatrics, The Second Hospital of Shandong University, Jinan, Shandong, China; 2 Department of Central Laboratory, The Second Hospital of Shandong University, Jinan, Shandong, China; 3 Department of General Practice, The Second Hospital of Shandong University, Jinan, Shandong, China; 4 Department of Pediatrics, Qilu Hospital of Shandong University, Jinan, Shandong, China

**Keywords:** bronchiolitis obliterans, diacetyl, fibrosis, ubiquitin C, airway injury

## Abstract

**Introduction:**

Bronchiolitis obliterans (BO) is an irreversible, chronic obstructive pulmonary disease with inflammation and fibrosis causing bronchiolar narrowing. Inhaling diacetyl (DA) can result in BO in humans.

**Methods:**

We aimed to investigate the relationship between fibrosis-related gene expression and ubiquitin C (UbC) regulation in a rat model of BO following a single DA instillation, by examining lung histopathology, UbC protein levels, and transcriptomic changes.

**Results:**

After DA exposure, rat bronchioles exhibited marked inflammation, increased collagen deposition, airway fibrosis, and obstruction. These changes were confirmed by histology and semi-quantitative image analysis. UbC protein levels were significantly elevated in a time-dependent manner. RNA sequencing revealed significant alterations in gene expression and enrichment of multiple molecular functions and biological processes in BO rats compared with controls, including pathways related to fibrosis formation and ubiquitin dysregulation. Quantitative PCR (qPCR) validation further confirmed the transcriptomic results, showing significant upregulation of Ube2t and Fap and downregulation of Cyp1a1, consistent with enhanced ubiquitin activity, fibroblast activation, and impaired oxidative stress regulation.

**Discussion:**

These findings indicate that DA instillation induces early BO-like changes, disrupts ubiquitin regulation, and increases UbC expression, potentially through oxidative stress–related mechanisms. A better understanding of ubiquitin regulation (particularly UbC) may provide novel molecular targets for therapeutic intervention in BO.

## 1 Introduction

Bronchiolitis obliterans (BO), also known as obliterative bronchiolitis, is a rare chronic obstructive lung disease characterized by airflow limitation resulting from bronchiolar stenosis and fibrosis. Lung function may gradually progressively deteriorate (Bar et al., 2014). Common causes of BO include inhalation of hazardous fumes, respiratory infections, connective tissue disorders, and complications following lung, heart, or bone marrow transplantation (Aguilar et al., 2016; Lynch et al., 2012). Diagnosis typically involves high-resolution computed tomography (CT), pulmonary function testing, and, in some cases, lung biopsy (Silva and Müller, 2008).

Industrial inhalants are another major cause of BO. About 2 decades ago, workers in a microwave popcorn factory exposed to butter flavoring vapors developed ‘popcorn worker’s lung,’ a form of BO (Schachter, 2002; Sauler and Gulati, 2012; Harber et al., 2006). Similar cases have been reported among workers in coffee and flavoring industries. The principal toxic agents identified were 2,3-butanedione (diacetyl, DA) and the structurally related 2,3-pentanedione, both of which disrupt airway epithelial integrity, alter protein homeostasis, and induce bronchiolar fibrosis in animal models (Harber et al., 2006; Kreiss, 2017; Hubbs et al., 2019). More recently, DA and related diketones have been detected in most commercial e-cigarette liquids, raising renewed concerns about their potential role in airway injury among vapers (Landman et al., 2019; Allen et al., 2015).

Ubiquitin is an evolutionarily conserved protein that covalently attaches to substrates via its C-terminal glycine residue, regulating protein degradation, localization, and signaling. The ubiquitination cascade involves three key enzymes: ubiquitin-activating (E1), ubiquitination-conjugating (E2), and ubiquitin ligases (E3) enzymes. Through post-translational modification, ubiquitination governs the ubiquitin-proteasome system (UPS), which controls proteolysis, mitosis, and cellular homeostasis (Dastsooz et al., 2019; Chaugule and Walden, 2016; Buetow and Huang, 2016; Spratt et al., 2014). K48-linked chains target proteins for proteasomal degradation, while K63-linked chains can mediate autophagy or signaling (Swatek and Komander, 2016). Dysregulation of ubiquitination has been implicated in various diseases, including cancer and fibrotic disorders (Perrotta et al., 2012; Villegas-Ruiz and Juarez-Mendez, 2016; Wang et al., 2017). Overexpression of E2-related genes such as UBE2C can lead to chromosomal instability and abnormal cell proliferation (van Ree et al., 2010). UbiquitinC (UbC) is one of four ubiquitin-encoding genes (Marinovic et al., 2002). It encodes a stress-inducible polyubiquitin precursor, highly sensitive to ultraviolet radiation, oxidative stress, and translational inhibition, and plays an important role in maintaining ubiquitin homeostasis (Radici et al., 2013; Ryu et al., 2007). Recent studies indicate that dysregulation of the ubiquitin–proteasome system contributes to pulmonary fibrosis and chronic lung injury by disrupting proteostasis and extracellular matrix turnover (Zhang et al., 2025; Lee et al., 2023; Chen et al., 2024). However, the specific role of UbC regulation in the fibrotic remodeling of BO remains unclear.

Therefore, this study aimed to investigate the relationship between fibrosis-related gene expression UbC activation in a rat model of diacetyl-induced BO. Histopathological evaluation, collagen semi-quantification, and UbC protein analysis were combined with transcriptomic profiling to elucidate the molecular mechanisms linking oxidative stress, ubiquitin dysregulation, and fibrosis. In addition, to validate key transcriptomic findings, representative genes related to ubiquitination (Ube2t), fibrosis (Fap), and oxidative metabolism (Cyp1a1) were selected for quantitative PCR (qPCR) analysis.

## 2 Materials and methods

### 2.1 Ethical approval

All data comply with scientific and ethical standards. All animal studies were approved by the Animal Ethics Committee of the Second Hospital of Shandong University, number KYU-2019(KJ)A-0160. Experimental animal management followed the Guide for the Care and Use of Laboratory Animals of the National Institutes of Health.

### 2.2 Construction of the BO rat model

Male Sprague–Dawley (SD) rats (n = 18, 250–300 g) were purchased from the Wuhan Experimental Animal Center (Wuhan, China). After weighing, they were randomly assigned to a DA instillation group (n = 9) and a control group (n = 9). All rats were observed daily, fed regularly and kept n a routine environment at a temperature of 24 °C ± 0.5 °C, and humidity of 55% ± 5%. DA (Sigma, United States) was diluted with physiological saline to a final concentration of 188 mg/mL. On day 1, rats were anesthetized with sodium pentobarbital (50 mg/kg, intraperitoneally) and DA (125 mg/kg) was instilled intratracheally as a single dose. The control group received an equal volume of normal saline. Three rats from each group were anesthetized, subjected to bronchoalveolar lavage fluid (BALF) collection, and then euthanized with an overdose of pentobarbital sodium (150 mg/kg, i.p.) on days 3, 5, and 7 after DA instillation for histopathological and molecular analyses.

### 2.3 Bronchoalveolar lavage fluid (BALF) measurement

BALF was collected by injection of 1 mL of sterile phosphate buffered saline (PBS) into the rats’ lungs, via the trachea, three times, and the total number of cells was counted with a blood cell counter. After centrifugation (for 10 min, at 500 g), the percentages of eosinophils, lymphocytes, and neutrophils were determined using microscopy.

### 2.4 Tissue sections and hematoxylin-eosin (H&E)

Lung tissue was dehydrated, and then sequentially immersed in three jars of paraffin (at 60 °C), and the wax-soaked tissue blocks, wrapped in paraffin, were sliced with a pathological microtome, placed in a 40 °C water bath, and baked at 60 °C for 3 h. The paraffin sections were dewaxed and stained with hematoxylin with eosin then added and then further dehydrated. Finally, the slices were air-dried under a fume hood. Neutral gum was added dropwise, slides were then covered with clean coverslips, and examined under a microscope. For collagen quantification, the percentage of collagen fiber area was measured from Masson-stained sections using ImageJ software (NIH, United States), and the mean of three randomly selected microscopic fields per sample was calculated.

### 2.5 Immunohistochemistry (IHC)

For IHC paraffin sections were dewaxed and EDTA antigen retrieval buffer was added, and heated at 98 °C for 20 min. Hydrogen peroxide 3% was added dropwise to inhibit endogenous peroxidase, and then goat serum added dropwise with incubation for 30 min. The primary antibodies, UbC (Rabbit, Proteintech, United States Cat# 10457-1-AP, dilution 1:1000) and GAPDH (Mouse, Proteintech, United States Cat# 60004-1-Ig, dilution 1:50000), were added and incubated overnight at 4 °C. The following day, horse radish peroxidase (HRP)-labelled goat anti-rabbit secondary antibody (ZSGB, China Cat# ZB-2301, 1:10000) and HRP-labelled goat anti-mouse secondary antibody (ZSGB, China Cat# ZB-2305, 1:10000) was added dropwise and incubated at 37 °C for 30 min. DAB chromogenic solution was added to each section which was then examined under a microscope. Harris hematoxylin counterstain was used, and after differentiation, sections were immersed in PBS to restore the blue color. After dehydration, until transparent, sections were air-dried, then sliced and sealed using neutral gum. The imaged under a confocal microscope (Olympus BX53)

### 2.6 Western blot (WB) analysis

Total protein was extracted from cells using lysis buffer for 30 min with centrifugation for protein quantification. The whole cell extract was mixed with Laemmli loading buffer, boiled for 5 min, and then subjected to SDS-PAGE. Proteins were transferred to a PVDF membrane and incubated with specific antibodies UbC (Rabbit, Proteintech, United States Cat# 10457-1-AP, dilution 1:1000) and GAPDH (Mouse, Proteintech, United States Cat# 60004-1-Ig, dilution 1:50000),. The membrane was washed with Tris-buffered saline and Tween-20 (TBST), and HRP-labelled secondary antibody, was added for 1 h at room temperature. Immune complexes were visualized using the ECL Western Blotting Detection Kit (ZSGB, China).

### 2.7 RT-qPCR analysis

Total RNA was extracted from lung tissues using the RNeasy Mini Kit (Qiagen United States), and RNA purity and concentration were determined with a NanoDrop 2000 spectrophotometer (Thermo Fisher Scientific, United States) by assessing the A260/A280 ratio. Complementary DNA (cDNA) was synthesized from 1 µg of total RNA using the High-Capacity cDNA Reverse Transcription Kit (Thermo Fisher Scientific, United States) according to the manufacturer’s instructions. Quantitative real-time PCR (RT-qPCR) was performed using 10 ng of cDNA per reaction with PowerTrack™ SYBR Green Master Mix (Thermo Fisher Scientific, United States) on a QuantStudio™ 7 Pro Real-Time PCR System (Applied Biosystems, United States). Each sample was analyzed in triplicate. The thermal cycling conditions were: initial denaturation at 50 °C for 2 min and 95 °C for 10 min, followed by 40 cycles of 95 °C for 15 s and 60 °C for 1 min. Relative gene expression was calculated using the 2^-ΔΔCt method, normalizing Ct values of target genes to the endogenous control (GAPDH) and comparing experimental to control groups. Primer sequences were designed using Primer-BLAST (NCBI) and synthesized by Sangon Biotech (Shanghai, China). Primer specificity was verified by melt-curve analysis showing a single amplification peak, and amplification efficiency ranged between 90% and 105%. Details of primer sequences are provided in Table 1.

**TABLE 1:** Primer sequences used for quantitative real-time PCR.

Gene symbol	Accession no.	Forward primer (5'→3′)	Reverse primer (5'→3′)	Product length (bp)	Annealing Tm (°C)	Source
Ube2t	NM_001108344.2	TGC​ACA​TGC​TAG​CCA​TCG​AA	CGG​ATC​TGT​GGT​GGC​TCA​AA	175	60	This study
Fap	NM_138850.1	CCA​GCT​GGG​AAT​ACT​ACG​CC	GGT​CAG​AGT​ACC​ACA​TCG​CC	153	60	This study
Cyp1a1	XM_006243150.4	CAG​AGG​TTG​GCC​ACT​TCG​AC	TAT​GCT​GAG​CAG​CTC​TTG​GTC	148	60	This study
Gapdh	NM_017008.4	GCA​TCT​TCT​TGT​GCA​GTG​CC	GGT​AAC​CAG​GCG​TCC​GAT​AC	122	60	Housekeeping gene

### 2.8 RNA isolation and expression analysis

RNA sequencing was performed on lung tissues collected at Day 7 after DA instillation (n = 3 per group). The RNA Nano 6000 Assay Kit for the Bioanalyzer 2100 System (Agilent Technologies, CA, United States) was used to assess RNA integrity. Samples were clustered on a cBot Cluster Generation System with TruSeq PE Cluster Kit v3-cBot-HS (Illumina) following the manufacturer’s instructions. Raw data in fastq format were first processed using fastp software. Differential gene expression analysis was performed on the specimens (3 biological replicates per time point) from the two groups using the DESeq2 R software package (1.20.0). Differential expression analysis was also performed using the EdgeR R package (3.22.5). The resulting P values were adjusted for false discovery rates using the Benjamini and Hochberg method. The clusterProfiler R package was used to implement gene ontology (GO) enrichment and Kyoto Encyclopedia of Genes and Genomes (KEGG) analysis of differentially expressed genes. P values less than 0.05 were considered to indicate significant enrichment of differentially expressed genes. Gene set enrichment analysis (GSEA) was performed from the webside (https://www.gsea-msigdb.org/gsea/index.jsp). The GO and KEGG datasets were independently used for GSEA. STRING was used to predict protein-protein interactions (https://string-db.org/).

### 2.9 Statistical analyses

Statistical analyses were performed using GraphPad Prism version 10.0 (GraphPad Software, United States). Differences between two groups were analyzed using the paired t-test, while comparisons among multiple groups were performed using one-way analysis of variance (ANOVA) followed by Tukey’s *post hoc* test. RNA-seq data were analyzed using the DESeq2 package, and the Benjamini–Hochberg false discovery rate (FDR) method was applied to correct for multiple testing. A P value <0.05 was considered statistically significant.

## 3 Results

### 3.1 Histopathologic changes characteristic of BO after DA instillation

During the experiment, rats in the control group remained healthy and showed no abnormal behavior. In contrast, rats in the DA-treated group exhibited signs of lethargy and reduced food intake by day 2, although respiration initially remained unaffected. Their general condition gradually improved over time; however, respiratory symptoms progressively worsened. By day 6, the remaining rats in the DA group developed marked dyspnea accompanied by intermittent coughing.

**FIGURE 1:**
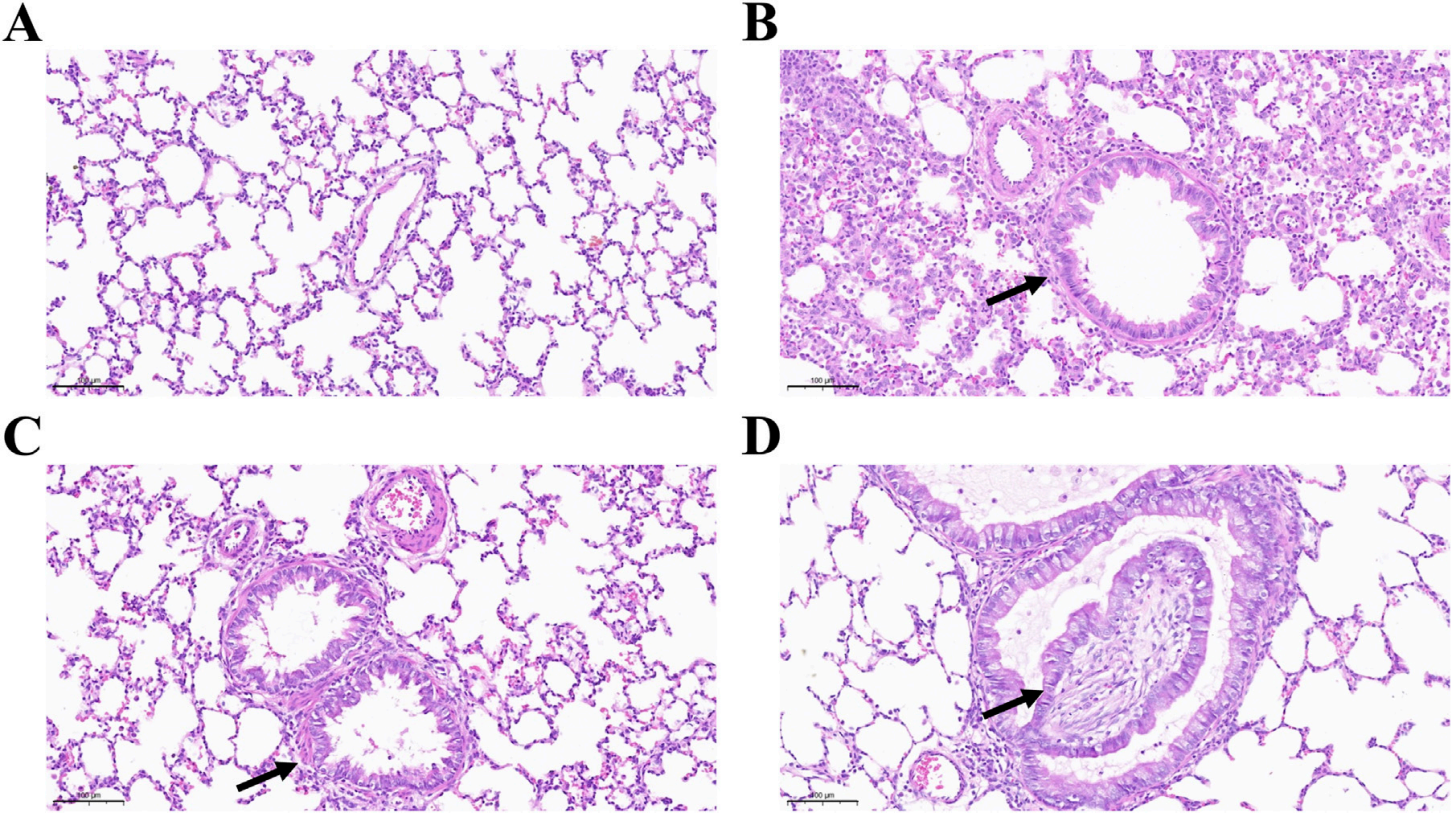
Intraluminal fibrosis and obliterative bronchiolitis after diacetyl (DA) instillation. **(A)** Normal bronchiolar epithelium and airway structure in the normal saline (NS) control group on day 7. **(B)** Bronchioles showing epithelial hyperplasia on day 3 after DA instillation (black arrow) (Scale bars: 100μm). **(C)** Bronchioles exhibiting concentric intramural fibrosis and luminal narrowing on day 5 after DA instillation (black arrow) (Scale bars: 100μm). **(D)** Extensive intraluminal fibrosis on day 7 after DA instillation, with almost complete airway obstruction, marked collagen deposition, and fibrotic remodeling (black arrow) (Scale bars: 100μm).

Figure 1 illustrates the histopathological progression of bronchiolitis obliterans (BO) and intraluminal fibrosis following DA exposure compared with saline controls. As the disease advanced, the loose connective tissue was progressively replaced by dense, mature collagen, leading to airway fibrosis. This resulted in loss of elasticity, impaired expansion and contraction, and diminished regenerative potential. Necrotizing fibrosis adjacent to the airway lumen caused varying degrees of tissue damage, and by day 7, the bronchiolar lumen was almost completely occluded by fibrotic tissue.

**FIGURE 2:**
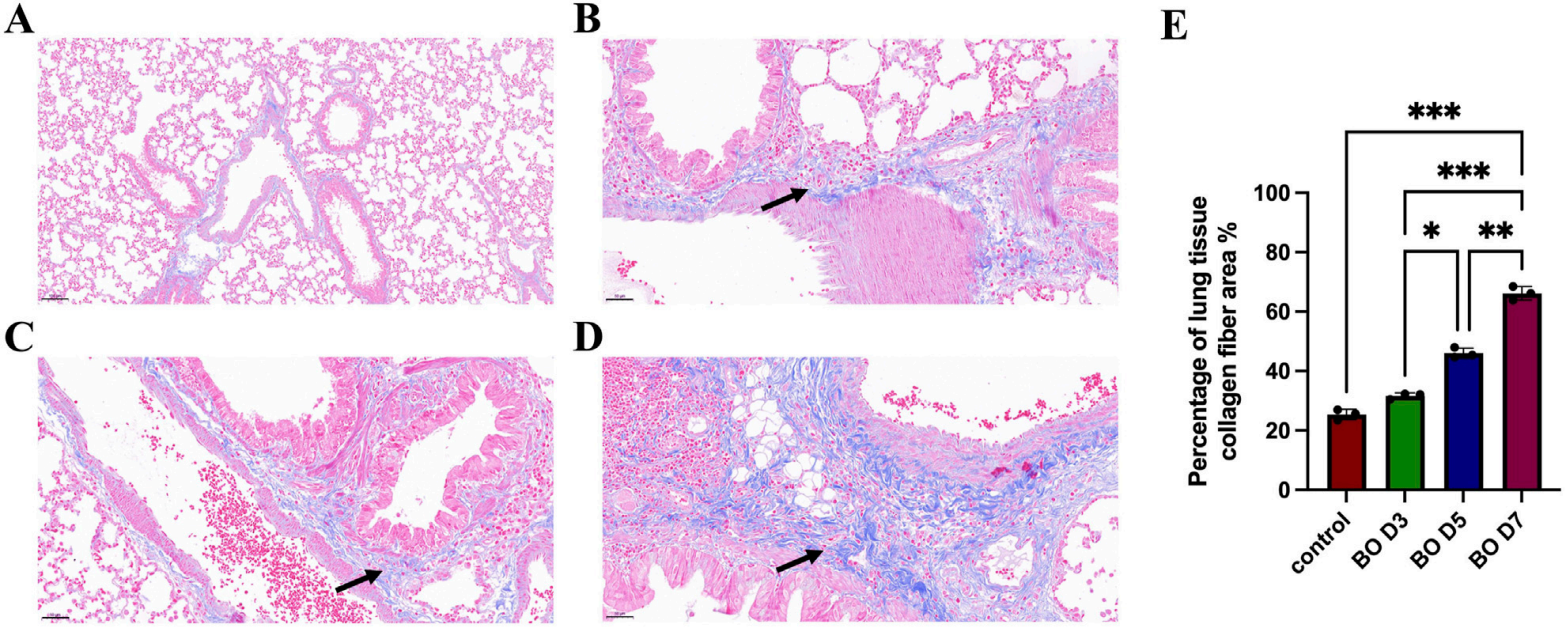
Peri-interstitial inflammation in bronchiolitis obliterans following diacetyl (DA) instillation. **(A)** Normal bronchioles from an NS control rat on day 7 showing intact airway epithelium and architecture (Scale bars: 100 μm). **(B)** On day 3 after DA instillation, bronchioles display peribronchiolar inflammation and epithelial regeneration with mild hyperplasia (black arrow) (Scale bars: 50 μm). **(C)** On day 5, bronchioles exhibit more extensive epithelial damage, tissue destruction, and dense infiltration of neutrophils and macrophages within the interstitium (black arrow) (Scale bars: 50 μm). **(D)** Masson’s trichrome staining reveals widespread fibroplasia and interstitial collagen deposition (black arrow), indicating progressive fibrosis and loss of normal bronchiolar structure (Scale bars: 50 μm). **(E)** Semi-quantitative analysis of collagen deposition. Data are presented as mean ± SD (n = 3 rats per group per time point). Statistical significance was determined by one-way ANOVA followed by Tukey’s *post hoc* test; P < 0.05 was considered statistically significant. *P < 0.05, **P < 0.01, and ***P < 0.001.

Figure 2 shows the development of peribronchial and interstitial inflammation. Compared with the saline group, the DA-treated rats displayed mild to moderate nodular hyperplasia of peribronchial lymphoid tissue on days 3 and 5, which became more diffuse by day 7. Linear hyperplasia and infiltration of mixed inflammatory cells were observed in the interstitial space. Masson’s trichrome staining revealed marked collagen deposition, suggesting that epithelial and mucosal injury contributed to the development of subsequent fibrosis. Quantitative analysis of collagen deposition showed a progressive increase in collagen fiber area percentage from day 3 to day 7 after diacetyl instillation, consistent with histological evidence of fibrosis.

### 3.2 Inflammatory cell response in BALF of BO following DA instillation

**FIGURE 3:**
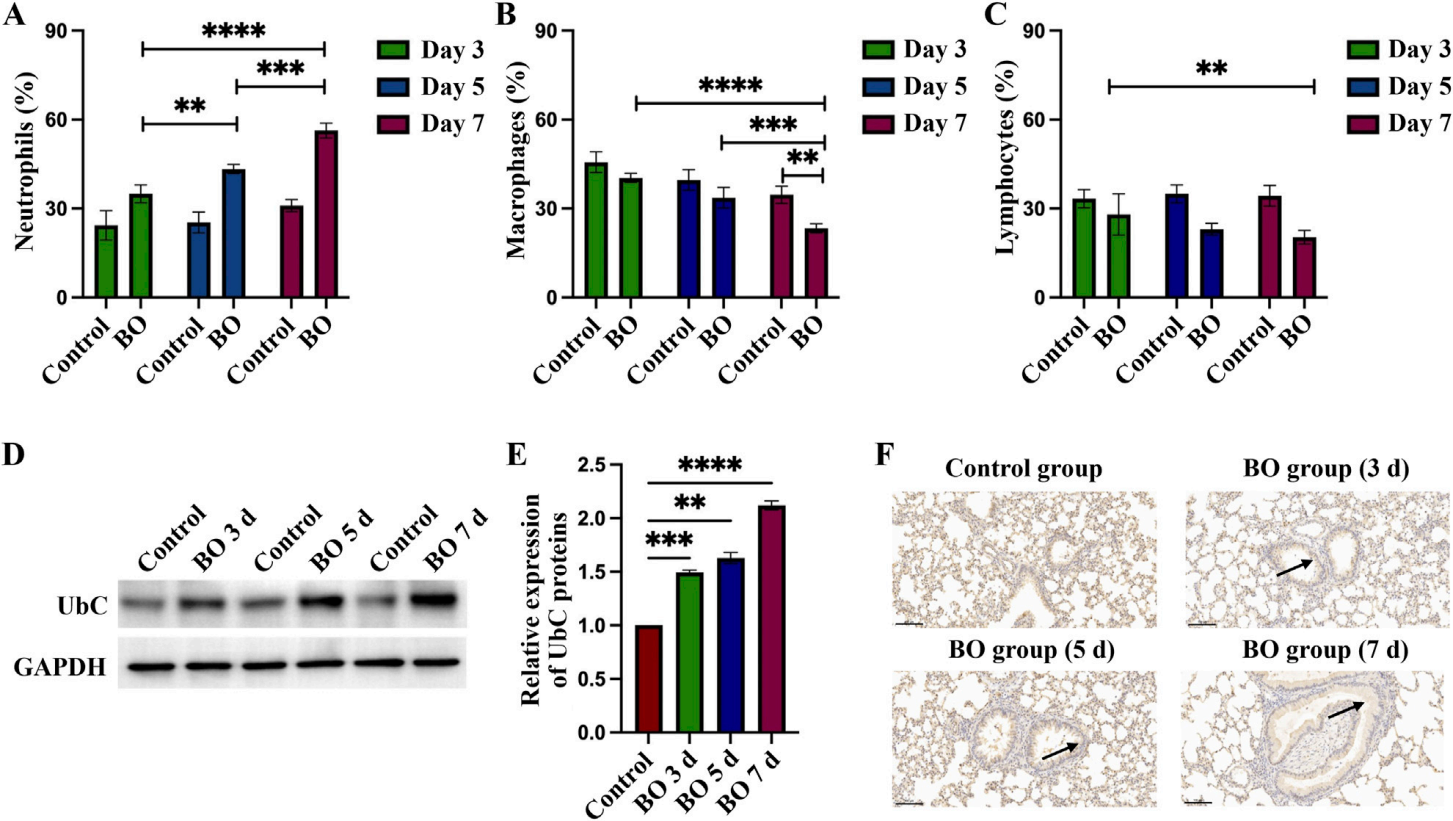
Differential cell percentages in BALF and expression of UbC in rats lungs after DA instillation. **(A–C)** Percentages of neutrophils, macrophages, and lymphocytes in bronchoalveolar lavage fluid (BALF) on days 3, 5, and 7 after DA instillation compared with saline controls. **(D)** Western blot analysis showing UbC protein expression at 3d, 5d, and 7d following DA instillation or saline treatment. **(E)** Quantification of UbC protein, normalized to total protein, expressed relative to saline control; **(F)** Immunohistochemical staining for UbC in lung sections demonstrating enhanced UbC expression in airway epithelial and peribronchial regions after DA exposure (black arrow) (Scale bars: 100 μm). Data are presented as mean ± SD (n = 3 rats per group per time point). Statistical significance was determined by one-way ANOVA followed by Tukey’s *post hoc* test; P < 0.05 was considered statistically significant. **P < 0.01, ***P < 0.001, and ****P < 0.0001.

BALF was examined to determine percentage changes in neutrophils, macrophages, and lymphocytes following DA exposure. Neutrophils increased significantly on days 3, 5, and 7, with statistical differences between the DA instillation and control groups. On day 7, the BALF neutrophilia was significantly higher than on days 5 and 3 after DA instillation (56.33 ± 2.52 vs. 43.33 ± 1.53 vs. 35.33 ± 4.93, Figure 3A). By contrast, the percentage of BALF macrophages gradually decreased after DA; on day 7 BALF macrophages were significantly lower than on days 5 and 3 (20.33 ± 1.53 vs. 33.67 ± 3.51 vs. 40.33 ± 1.53, Figure 3B). BALF lymphocytes were significantly decreased on day 7 after DA instillation compared to saline control (20.33 ± 2.31 vs. 34.33 ± 3.51) but there were no significant differences on days 3 and 5 (Figure 3C).

### 3.3 Increased expression of UbC in BO after DA instillation

After instillation of DA, UbC protein expression on WB of rat lungs increased significantly, compared to the saline control group, on days 3, 5, and 7 (1.49 ± 0.02 vs. 1.63 ± 0.05 vs. 2.1 2 ± 0.04, Figures 3D,E). Increased UbC protein expression was observed on lung tissue sections in the intrapulmonary airways, gradually increasing after DA instillation; but was seen only rarely in controls (Figure 3F).

### 3.4 Identification of differentially expressed genes in BO after DA instillation

**FIGURE 4:**
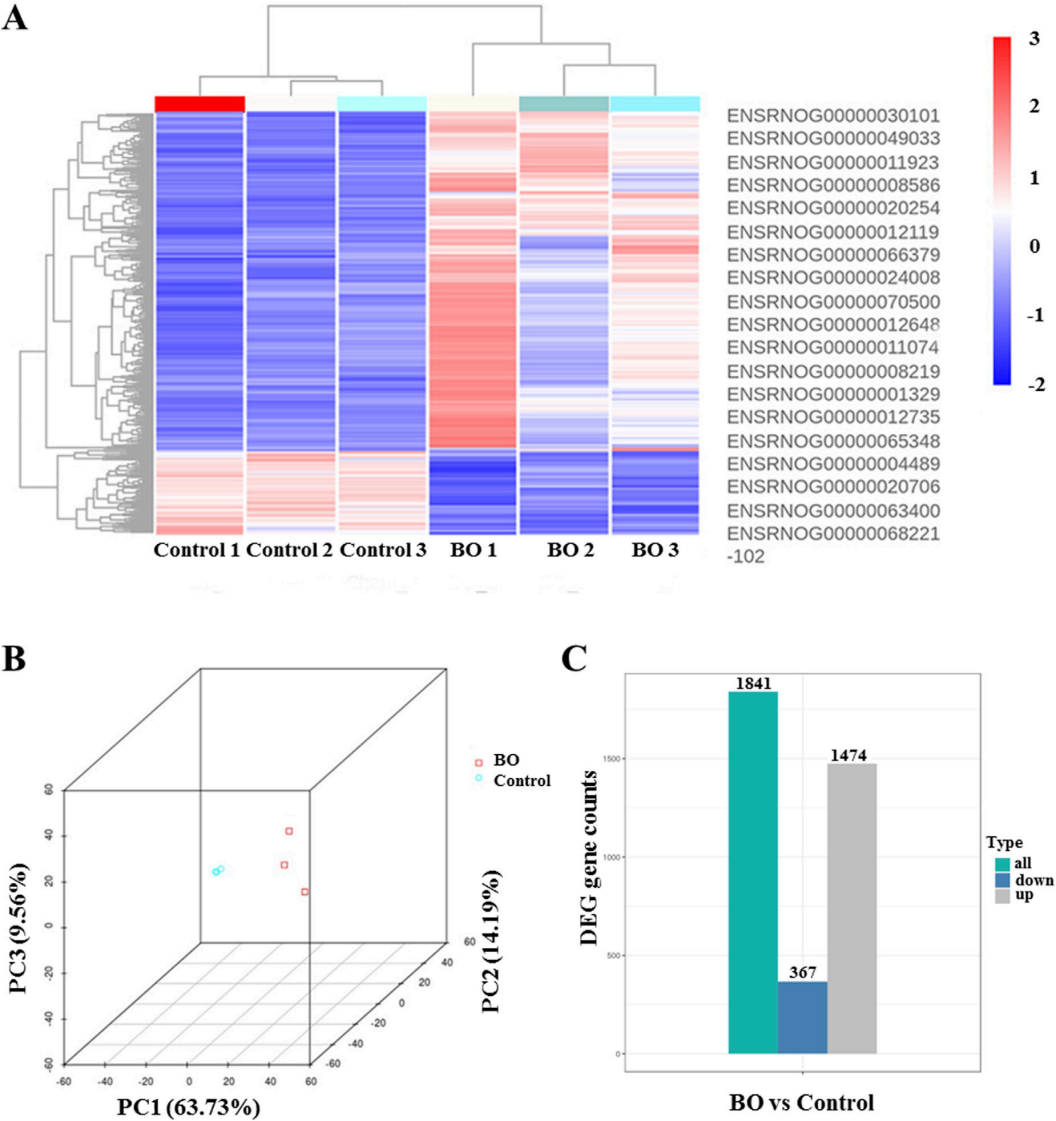
Differential gene expression profiles in bronchiolitis obliterans (BO) rats following diacetyl (DA) instillation. Transcriptomic profiling was performed on Day 7 lung tissues from DA-treated and saline control rats (n = 3 per group). **(A)** Heat map showing representative differentially expressed genes (DEGs) between BO and control groups. Red indicates upregulated genes, and blue indicates downregulated genes. **(B)** Principal component analysis (PCA) plot demonstrating clear separation between DA-treated and control samples, indicating consistent transcriptomic alterations induced by DA exposure. **(C)** Overall distribution of differentially expressed genes, summarizing the total number of upregulated and downregulated genes identified in BO compared with controls. Differential expression was analyzed using DESeq2 (padj ≤0.05, |log_2_FoldChange| ≥ 1.0).

Transcriptomic profiling was performed using Day 7 samples, which exhibited the most representative histopathological and biochemical features of BO. mRNA sequencing was employed to investigate transcriptomic alterations induced by DA exposure. Principal component analysis (PCA) revealed distinct transcriptomic separation between the DA-treated and saline control groups (Figure 4B). Hierarchical clustering heat maps demonstrated consistent expression patterns within each group (Figure 4A). Compared with the saline control group, transcriptomic profiling of BO rats revealed the overall distribution of expressed genes, with 1,474 genes showing increased expression, 367 showing decreased expression, and the remainder exhibiting no significant change (Figure 4C).

**FIGURE 5:**
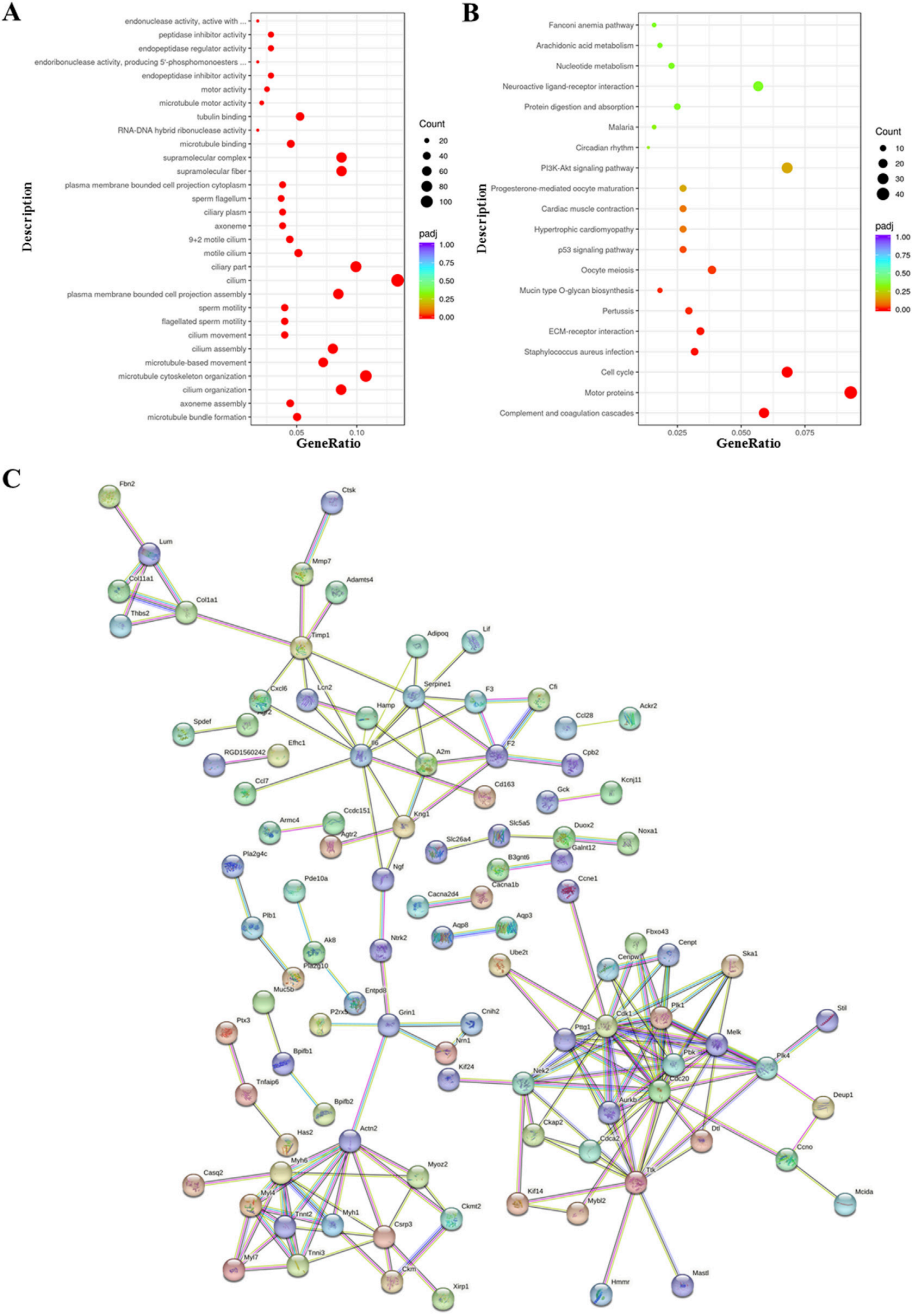
Functional enrichment analysis of differentially expressed genes in bronchiolitis obliterans (BO) following diacetyl (DA) instillation. **(A)** Gene Ontology (GO) enrichment analysis, showing the 30 most significantly enriched biological terms. **(B)** Kyoto Encyclopedia of Genes and Genomes (KEGG) pathway analysis, highlighting the top 20 enriched signaling pathways. **(C)** STRING protein–protein interaction network, in which nodes represent upregulated proteins and connecting lines indicate functional associations. For all enrichment analyses, genes with |log2FoldChange| ≥ 2 and adjusted p < 0.05 were included. In the bubble plots, the x-axis represents the gene ratio, bubble size indicates the number of enriched genes, and bubble color corresponds to–log10 (adjusted p-value).

Functional enrichment analysis of DEGs was performed using the ClusterProfiler R package. Gene Ontology (GO) analysis revealed significant enrichment of genes associated with microtubule motor activity, tubulin binding, microtubule organization, and ciliary structure and movement, including “supramolecular fiber,” “motile cilium,” and “microtubule bundle formation” (Figure 5A). Kyoto Encyclopedia of Genes and Genomes (KEGG) pathway analysis further showed enrichment of genes involved in neuroactive ligand–receptor interaction, protein digestion and absorption, PI3K–Akt signaling, cardiac muscle contraction, hypertrophic cardiomyopathy, ECM–receptor interaction, cell cycle regulation, complement and coagulation cascades, and *Staphylococcus aureus* infection (Figure 5B).

Protein–protein interaction (PPI) network analysis using the STRING database demonstrated strong consistency with the GO and KEGG enrichment results, highlighting interconnected molecular pathways contributing to fibrosis and airway remodeling (Figure 5C).

### 3.5 Fibrosis-associated and ubiquitin-related gene expression in BO rats after diacetyl exposure

Differential gene analysis and GSEA were performed to explore fibrosis and ubiquitin-associated pathways in bronchiolitis obliterans (BO) rats. A total of 497 genes were upregulated and 81 were downregulated in the DA group compared with saline controls (Padj ≤0.01, |Log_2_FoldChange| ≥ 2.0). The top differentially expressed genes identified on Day 7 are listed in Supplementary Table S1.

**FIGURE 6:**
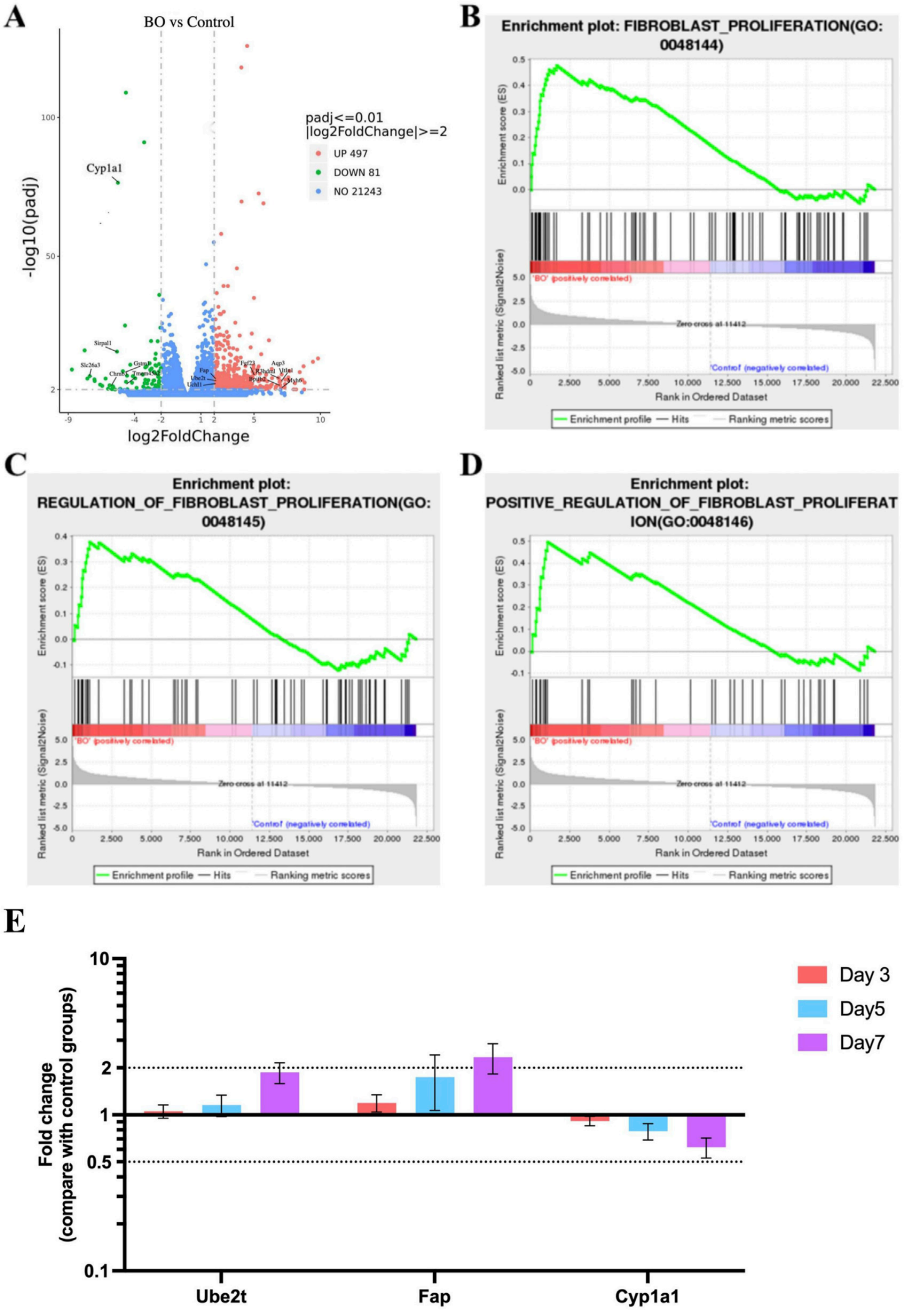
Fibrosis-related gene activation and ubiquitin pathway dysregulation in DA-induced bronchiolitis obliterans. **(A)** Volcano plot showing differentially expressed genes (DEGs) in the BO and saline control groups. Red and green dots represent significantly upregulated and downregulated genes, respectively (padj <0.01, |log_2_FoldChange| ≥ 2).The top genes with the greatest |log_2_FoldChange| are labeled on the plot. **(B)** Enrichment of the Fibroblast proliferation gene set. **(C)** Enrichment of the Regulation of fibroblast proliferation gene set. **(D)** Enrichment of the Positive regulation of fibroblast proliferation gene set. **(E)** Quantitative PCR validation of Ube2t, Fap, and Cyp1a1 genes. Data are shown as mean ± SD (n = 3 rats per group, each sample run in triplicate). Genes with |log_2_FoldChange| ≥ 2 and adjusted p < 0.05 were defined as significantly differentially expressed. Significance was determined by false discovery rate (FDR) < 0.25 and adjusted p < 0.05 after 1,000 permutations. Positive enrichment scores (NES >0) indicate upregulation in DA-treated lungs, while negative scores (NES <0) indicate downregulation.

**FIGURE 7:**
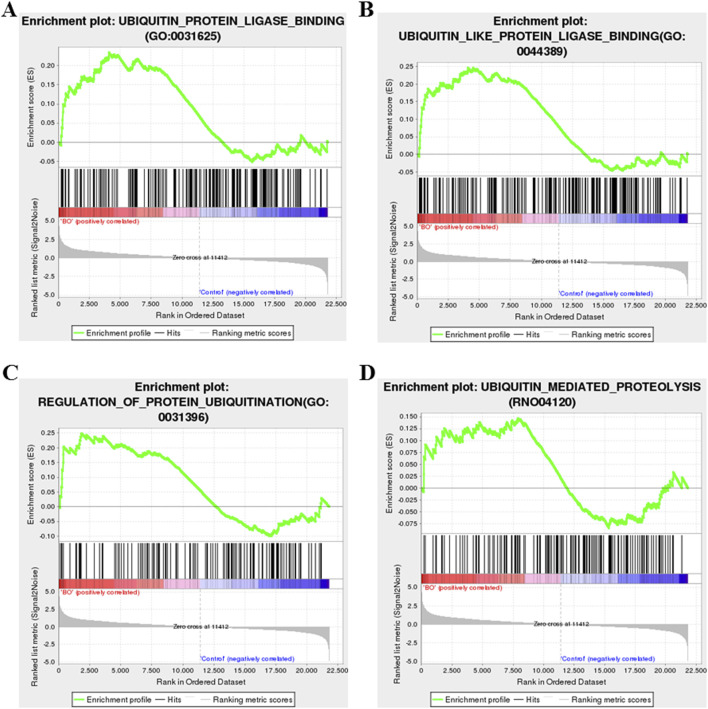
Gene set enrichment analysis (GSEA) of ubiquitin-related pathways in DA-induced BO. **(A)** Enrichment of the ubiquitin–protein ligase activity gene set. **(B)** Enrichment of the ubiquitin-like protein ligase binding gene set. **(C)** Enrichment of the regulation of protein ubiquitination gene set. **(D)** Enrichment of the ubiquitin-mediated proteolysis gene set. Significance was determined by false discovery rate (FDR) < 0.25 and adjusted p < 0.05 after 1,000 permutations. Positive enrichment scores (NES >0) indicate upregulation in DA-treated lungs, while negative scores (NES <0) indicate downregulation.

Among the most upregulated genes were Bpifb2, Myh6, Aqp3, Itln1, R3hdml, while Cyp1a1, Slc26a3, Sirpa1, Chrnb3, and Gstm1 were among the most downregulated (Figure 6A). In addition, genes related to ubiquitin regulation, including Ube2t and Uchl1, fibrosis-related gene including Fap, showed marked upregulation, suggesting activation of ubiquitin–proteasome and fibrosis-associated signaling. Notably, Cyp1a1—involved in oxidative metabolism—was markedly suppressed, consistent with oxidative stress–induced disruption of detoxification pathways. GSEA revealed significant enrichment of fibroblast proliferation–related gene sets, including “fibroblast proliferation,” “regulation of fibroblast proliferation,” and “positive regulation of fibroblast proliferation” (Figures 6B–D). In parallel, gene sets related to ubiquitin-protein ligase activity, ubiquitin-like protein ligase binding, regulation of protein ubiquitination, and ubiquitin-mediated proteolysis were significantly enriched in the BO group (Figures 7A–D), supporting a central role of ubiquitin dysregulation in fibroblast activation and airway fibrosis.

To validate these transcriptomic findings, qPCR was performed for three representative genes. Consistent with RNA-seq data, Ube2t and Fap were significantly upregulated in a time-dependent manner, while Cyp1a1 was downregulated following DA exposure (P < 0.05, Figure 6E). These results support that DA-induced airway injury involves activation of ubiquitin-related pathways and fibroblast proliferation, contributing to fibrotic airway remodeling.

The results demonstrate that a single intratracheal instillation of diacetyl induces a cascade of pathological changes characterized by inflammatory cell infiltration, progressive collagen deposition, and airway fibrosis expecially day 7. These histological alterations were accompanied by increased expression of UbC protein and significant transcriptomic changes involving fibrosis-related and ubiquitin-associated genes. The integration of these findings suggests that diacetyl exposure triggers both fibrogenic activation and disruption of ubiquitin-mediated proteostasis, processes that may collectively contribute to the development and persistence of bronchiolitis obliterans. To further interpret these results and their mechanistic implications, we expanded the following Discussion section.

## 4 Discussion

BO is a rare condition in which airway inflammation and peribronchial fibrosis led to bronchiolar stenosis, concentric narrowing and even complete occlusion. Its histopathologic features include epithelial disruption, bronchiolar smooth muscle hypertrophy, peribronchiolar inflammation and granulation tissue, fibrous tissue hyperplasia, scarring of distal airways and luminal obstruction. Small airways obstruction leads to hyperinflation (air trapping), patchy atelectasis, with a reduction in blood vessel volume and number, resulting in impaired ventilation and hypoxemia (Kerger and Fedoruk, 2015; Krishna et al., 2024). DA is water-soluble and is readily absorbed through the mucous membranes of the nose, pharynx, larynx and trachea producing an acute inflammatory response and potential damage (Gloede et al., 2011).

Various animal studies have demonstrated that inhalation or tracheal instillation of diacetyl (DA) and its analog 2,3-pentanedione induces airway injury and fibrosis similar to those observed in human bronchiolitis obliterans. Both rat and mouse models have been widely used to explore the mechanisms underlying distal airway inflammation and bronchiolar remodeling (Morgan et al., 2012; Morgan et al., 2008). High concentrations of inhaled DA lead to marked bronchiolar inflammation, epithelial sloughing, and progressive fibrotic changes in the small airways (Krishna et al., 2024). A single intratracheal dose of DA has been shown to reproduce the cardinal histopathological features of BO in rats, including epithelial injury, peribronchial inflammation, and luminal fibrosis (Kreiss, 2014). In the present study, we employed this acute high-dose intratracheal model to achieve reproducible epithelial damage and early fibrotic remodeling, which were consistent with published animal models (Morgan et al., 2012; Morgan et al., 2008; Palmer et al., 2011; Flake and Morgan, 2017) and the pathological manifestations of human BO. While occupational and vaping exposures are typically chronic and lower in concentration, both acute and chronic DA exposures activate similar downstream pathways involving oxidative stress, epithelial injury, ubiquitin dysregulation, and fibroblast-driven fibrosis. This acute model therefore effectively captures the early molecular events, particularly UbC upregulation and disturbance of ubiquitin homeostasis, that initiate the fibrotic remodeling characteristic of BO.

Ubiquitin is encoded by UbC, ubiquitin B (UBB), UBA52, and RPS27A; polyubiquitin-C is another source of ubiquitin. Polyubiquitin-C controls a multitude of biological functions. Under physiological circumstances, it assists maintenance of homeostatic levels of ubiquitin which is stimulated in response to heat shock, oxidative stress, and translational damage (Ki et al., 2010; Snyder and Silva, 2021). UbC is activated during gene transcription and stress and provides excess ubiquitin to remove damaged/unfolded proteins (Ryu et al., 2007). Polyubiquitin-C is involved in a variety of biological processes, including innate immunity, DNA repair, and kinase activity (Rajsbaum et al., 2014; Rajsbaum and García-Sastre, 2014; Tsirigotis et al., 2001; Pickart et al., 2004). In mouse embryonic fibroblasts, the UbC gene was investigated, and homozygous deletion was found to reduce cellular ubiquitin levels and viability under oxidative stress (Ryu and Ryu, 2011). Repetitive inhaled DA was shown to promote ubiquitin proteasomal stress and UbC protein expression (Wang et al., 2021). Following DA inhalation, extensive accumulation of total ubiquitin, K63-ubiquitin, and scaffolding protein sequestome-1 (SQSTM1) puncta in respiratory epithelial cells was demonstrated, indicating severe damage to proteins in the airways. The colocalization of ubiquitin with SQSTM1 and airway epithelial cell lysosomes implies that DA-induced airway damage involves extensive autophagy (Hubbs et al., 2016). We found UbC protein levels were significantly increased 7 days after a single DA administration, suggesting a stress-induced ubiquitin response associated with oxidative injury in BO rats. UbC upregulation appears to represent a stress-induced activation of the ubiquitin–proteasome system, contributing to early regulatory events in fibrotic remodeling. Rather than implying a direct causal role, these findings indicate that UbC may serve as a key adaptive regulator linking oxidative stress, proteostasis imbalance, and fibroblast activation.

To further elucidate the molecular mechanisms underlying these processes, RNA sequencing revealed distinct gene expression profiles in BO rats compared with controls. GO enrichment analysis identified significant alterations in genes involved in microtubule assembly, ciliary function, and extracellular matrix organization, all of which are implicated in fibrosis development. KEGG pathway analysis showed enrichment in PI3K-Akt signaling, ECM-receptor interactions, cell cycle regulation and coagulation pathways, consistent with fibroblast activation and immune dysfunction. GSEA demonstrated enrichment of genes related to fibroblast proliferation, ubiquitin ligase activity, protein ubiquitination, and proteostasis regulation, linking ubiquitin pathway disturbance with fibrotic remodeling.

qPCR validation further confirmed these transcriptomic findings, showing significant upregulation of Ube2t and Fap, and downregulation of Cyp1a1, consistent with enhanced ubiquitin activity, fibroblast activation, and impaired oxidative stress regulation. These genes collectively support the hypothesis that DA exposure simultaneously triggers proteotoxic stress and fibrotic signaling cascades, promoting irreversible airway remodeling.

Taken together, our findings provide a coherent and mechanistically novel model of diacetyl (DA)-induced bronchiolitis obliterans. By combining histopathology, protein analysis, and transcriptomic profiling, we demonstrated for the first time that a single high-dose DA exposure is sufficient to trigger early, reproducible features of bronchiolar fibrosis in rats. Following DA exposure, airway epithelial injury and inflammatory infiltration initiate a fibrotic cascade characterized by collagen deposition and fibroblast activation. At the molecular level, our data reveal a previously unrecognized disturbance of the ubiquitin–proteasome system—marked by UbC upregulation and altered expression of Ube2t and Uchl1—which appears to link oxidative stress with fibrotic remodeling. These findings integrate structural and molecular evidence into a unified pathogenic framework, highlighting the central role of ubiquitin dysregulation in the early initiation of airway fibrosis and providing new mechanistic insight that may inform future therapeutic strategies for BO.

Although this study provides new evidence linking fibrosis-related gene activation and ubiquitin dysregulation to diacetyl-induced bronchiolitis obliterans, several aspects warrant further exploration. Our transcriptomic analysis was conducted on whole-lung tissue, and cell-type–specific contributions to UbC induction and fibrosis require more precise delineation. The observed association between UbC upregulation, fibroblast activation, and extracellular matrix remodeling strongly suggests a central regulatory role for UbC in airway fibrosis. In addition, as the current model reflects early fibrotic remodeling, extending observations to later time points may clarify whether these ubiquitin-related alterations persist and drive chronic airway obstruction. This experiment was conducted as a single exposure series, the model has demonstrated highly consistent histological and transcriptomic outcomes across animals, supporting reproducibility. Future studies will incorporate independent biological replicates to validate the robustness of these findings and examine UbC-related responses across extended time frames. Overall, these findings provide a robust molecular foundation for understanding UbC-mediated pathways in fibrotic airway injury and establish a basis for future mechanistic and therapeutic investigations.

Collectively, these efforts will refine the mechanistic framework proposed here and may ultimately contribute to the development of novel therapeutic strategies targeting ubiquitin regulation in bronchiolitis obliterans.

## 5 Conclusion

In conclusion, a single intratracheal instillation of diacetyl (DA) induced characteristic pathological features of bronchiolitis obliterans in rat lungs, including epithelial injury, inflammation, and progressive fibrosis. These structural alterations were accompanied by disrupted ubiquitin regulation and a marked increase in UbC protein expression, suggesting a stress-induced activation of the ubiquitin–proteasome system linked to oxidative injury. The present findings highlight the pivotal role of ubiquitin dysregulation in the early stages of airway fibrosis and provide a molecular foundation for exploring UbC-targeted interventions in the prevention and treatment of bronchiolitis obliterans.

## Data Availability

All data generated or analyzed during this study are included in this published article and its supplementary information files. The raw sequence data reported in this paper have been deposited in the Genome Sequence Archive (Genomics, Proteomics & Bioinformatics 2021) in National Genomics Data Center (Nucleic Acids Res 2021), China National Center for Bioinformation/Beijing Institute of Genomics, Chinese Academy of Sciences (GSA: CRA018830) that are publicly accessible at https://ngdc.cncb.ac.cn/gsa.

## Supplementary material

SUPPLEMENTARY TABLE S1 The top 30 differentially expressed genes (DEGs) identified at Day 7 (|log_2_FC| ranked), as RNA-seq was performed exclusively at this time point.

## Abbreviations

BALF, Bronchoalveolar lavage fluid; BO, Bronchiolitis obliterans; Cyp1a1, Cytochrome P450 family 1 subfamily A member 1; involved in oxidative metabolism; DA, Diacetyl; DEGs, Differentially expressed genes; E2, Ubiquitin-conjugating enzyme; Fap, Fibroblast activation protein; marker of fibrosis; GAPDH, Glyceraldehyde-3-phosphate dehydrogenase; GSEA, Gene set enrichment analysis; GO, Gene ontology; H&E, Hematoxylin-eosin; HRP, Horseradish peroxidase; IHC, Immunohistochemistry; KEGG, Kyoto Encyclopedia of Genes and Genomes; PCA, Principal component analysis; PPI, Protein–protein interaction; qPCR, Quantitative polymerase chain reaction; SD, Sprague–Dawley; SQSTM1, Sequestosome-1 (p62) scaffolding protein; UbC, Ubiquitin C; Ube2t, Ubiquitin-conjugating enzyme E2T; WB, Western blot.
